# Ubiquitin-Related Modifier 1 (URM-1) Modulates Cx43 in Breast Cancer Cell Lines

**DOI:** 10.3390/ijms24032958

**Published:** 2023-02-03

**Authors:** Layal El-Hajjar, Jessica Saliba, Mario Karam, Abdullah Shaito, Hiba El Hajj, Marwan El-Sabban

**Affiliations:** 1 Department of Anatomy, Cell Biology and Physiological Sciences, Faculty of Medicine, American University of Beirut, Beirut P.O. Box 11-0236, Lebanon; 2 Department of Biology, Faculty of Sciences, Lebanese University, Beirut P.O. Box 90656, Lebanon; 3 Department of Public Health, Faculty of Health Sciences, University of Balamand, Beirut P.O. Box 100, Lebanon; 4 Biomedical Research Center, Qatar University Doha, Doha P.O. Box 2713, Qatar; 5 Department of Experimental Pathology, Immunology and Microbiology, Faculty of Medicine, American University of Beirut, Beirut P.O. Box 11-0236, Lebanon

**Keywords:** Cx43, URM-1, post-translational modification, breast cancer, epithelial-to-mesenchymal transition

## Abstract

Gap-junction-forming connexins are exquisitely regulated by post-translational modifications (PTMs). In particular, the PTM of connexin 43 (Cx43), a tumor suppressor protein, regulates its turnover and activity. Here, we investigated the interaction of Cx43 with the ubiquitin-related modifier 1 (URM-1) protein and its impact on tumor progression in two breast cancer cell lines, highly metastatic triple-negative MDA-MB-231 and luminal breast cancer MCF-7 cell lines. To evaluate the subsequent modulation of Cx43 levels, URM-1 was downregulated in these cells. The transcriptional levels of epithelial-to-mesenchymal transition (EMT) markers and the metastatic phenotype were assessed. We demonstrated that Cx43 co-localizes and interacts with URM-1, and URMylated Cx43 was accentuated upon cellular stress. The significant upregulation of small ubiquitin-like modifier-1 (SUMO-1) was also observed. In cells with downregulated URM-1, Cx43 expression significantly decreased, and SUMOylation by SUMO-1 was affected. The concomitant expression of EMT markers increased, leading to increased proliferation, migration, and invasion potential. Inversely, the upregulation of URM-1 increased Cx43 expression and reversed EMT-induced processes, underpinning a role for this PTM in the observed phenotypes. This study proposes that the URMylation of Cx43 in breast cancer cells regulates its tumor suppression properties and contributes to breast cancer cell malignancy.

## 1. Introduction

Connexins are integral membrane proteins that form gap junctions, enabling the direct cytoplasmic exchange of ions and low-molecular-weight metabolites between adjacent cells [1]. Connexins facilitate direct intercellular communication, which is a pivotal feature for the development, function, and homeostasis of tissues and organs [2,3]. An increasing number of gap-junction-independent functions are also attributed to these proteins [4]. Connexins are extensively regulated at the transcriptional, post-transcriptional, and post-translational levels, leading to the modulation of their trafficking, stability, and activity [5]. Most connexins harbor multiple consensus sites for phosphorylation, S-nitrosylation, ubiquitylation, and SUMOylation. Among connexin proteins, Cx43 is the most widely studied connexin. We have previously demonstrated the involvement of Cx43 in the metastatic potential of triple-negative breast cancer [6,7,8,9,10] and in inflammation [6,11]. PTMs play an important role in protein targeting, turnover, the regulation of chromatin structure, DNA metabolism, and genome stability. Cx43 activity, turnover, subcellular localization, and protein–protein interactions are regulated by ubiquitylation and SUMOylation, two documented PTMs [12,13,14,15], which exert their effects in a spatiotemporal manner [14]. Consequently, the role of Cx43 in its different PTM forms has been extensively studied in cancer progression and metastasis [16,17,18,19,20,21,22,23].

URMylation, the covalent conjugation of proteins by ubiquitin-related modifier 1 (URM-1), is a more recently described PTM [12], whose functional implications are being unraveled. URM-1 acts as a protein modifier, as well as a carrier of sulfur in the thiolation that takes place during the folding of eukaryotic transfer RNAs [13,14]. URM-1 is conserved from yeast to humans [15] and has been described in several experimental models. In *Saccharomyces cerevisiae*, URM-1 is involved in nutrient sensing and budding [24]. In *Drosophila melanogaster*, URM-1 regulates the oxidative stress and c-Jun N-terminal kinase (JNK) pathways [25,26]. In the fly model, the functional clustering of URM-1-associated proteins was implicated in cellular stress pathways, such as immunological threats and DNA damage [26]. In mammalian cells, protein URMylation increases in response to oxidative stress [27], and this PTM was more recently reported in oncoproteins [25]. In that sense, URMylation of Tax, a highly post-translationally modified oncoprotein in adult T-cell leukemia/lymphoma (ATL) was described [25] and regulates its shuttling and the expression of nuclear factor kappa B (NF-κB) target genes in human ATL cell lines [28].

Here, we investigate the status and functional consequences of URMylation in the regulation of Cx43 in a triple-negative breast cancer cell line, MDA-MB-231, and in a luminal breast cancer cell line, MCF-7. We demonstrate that Cx43 co-localizes and interacts with URM-1. The downregulation of URM-1 results in a sharp decrease in Cx43 expression, leading to an enhanced malignant phenotype. This is demonstrated by the increase in proliferation and migration and the increased expression of EMT markers and invasion potential. Conversely, the upregulation of URM-1 upregulated Cx43 expression and reversed EMT-induced processes, highlighting the implication of this PTM in Cx43 tumor suppression properties.

## 2. Results

### 2.1. Cx43 Interacts with Ubiquitin, URM-1, and SUMO-1

**Figure 1:**
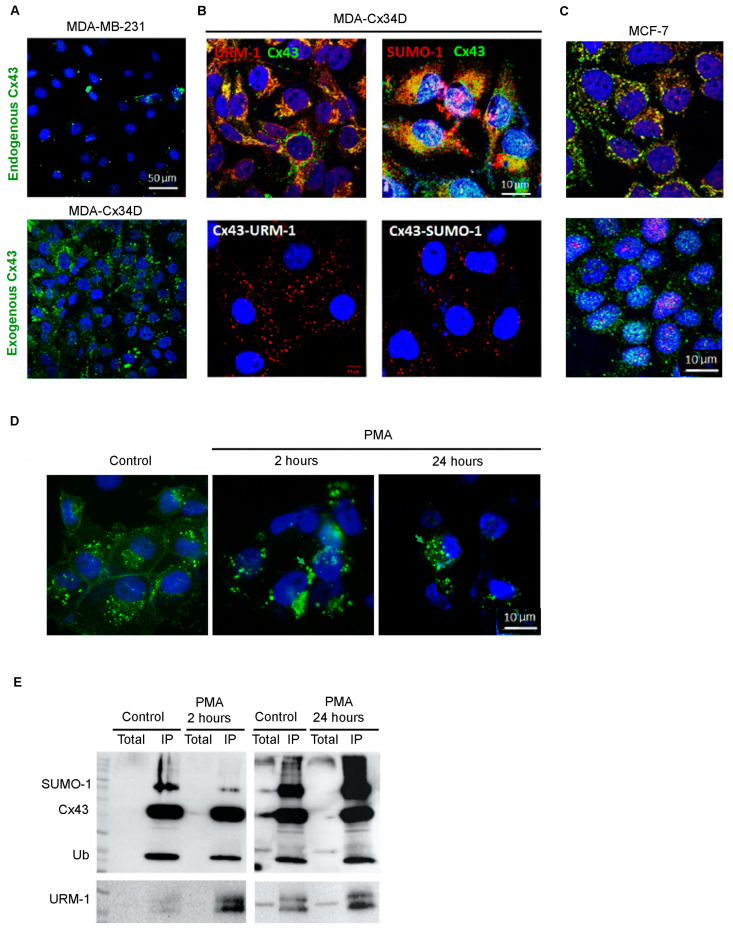
Cx43 interacts with URM-1 and SUMO-1 in breast cancer cells. (**A**) MDA-MB-231 cells express low levels of endogenous Cx43, which was upregulated in MDA-Cx34D cells. (**B**) Immunofluorescence assay (upper panel) and Duolink^®^ in situ proximity ligation assay (lower panel) were performed in MDA-Cx43D cells. Cx43 co-localizes and interacts with URM-1 and SUMO-1 primarily in the cytoplasmic compartment. (**C**) Immunofluorescence assay shows co-localization of Cx43 with URM-1 and SUMO-1 in MCF-7 cells. (**D**) Immunofluorescence assay shows annular junction formation (green arrows) in MDA-Cx43D upon treatment for 2 h and 24 h post-treatment with PMA. (**E**) MDA-Cx43D cells were treated with PMA, and Cx43 was evaluated by fluorescence. Micrographs show that PMA resulted in the internalization of Cx43. (**E**) Immunoprecipitation assay showed that URM-1 co-precipitates with Cx43 as early as 2 h post-PMA treatment, and that SUMO-1 heavily interacts with Cx43 24 h post-PMA treatment. All panels are representative of at least three experiments. DAPI is shown in blue, SUMO-1 and URM-1 are shown in red, and Cx43 is shown in green.

The triple-negative breast cancer cell line MDA-MB-231 expresses low levels of the tumor suppressor protein Cx43 (Figure 1A, upper panel). We upregulated the expression level of this key connexin and generated MDA-Cx43D cells (Figure 1A, lower panel), and we showed that URM-1 and SUMO-1 co-localize with Cx43 (Figure 1B, upper panel). URM-1 exhibits a pattern similar to other protein modifiers, notably Ub and SUMO-1, but not SUMO-2/3 (Figure 1B, panel; Supplementary Figure S1A). The proximity ligation assay (PLA; Duolink^®^) confirmed the co-localization and interaction of Cx43 with URM-1, ubiquitin (Ub), and SUMO-1, but not SUMO-2/3 (Figure 1B; Supplementary Figure S1A). Similarly, in MCF-7 cells, URM-1, Ub, and SUMO-1 are also expressed and co-localize with Cx43 (Figure 1C; Supplementary Figure S1B).

To further confirm the PLA data, immunofluorescence and co-immunoprecipitation assays of Cx43 and URM-1 were conducted. For that purpose, MDA-Cx43D cells were exposed to the tumor-promoting phorbol ester (PMA), which, upon Protein Kinase C activation, regulates the trafficking, assembly, degradation, and channel gating of Cx43 gap junctions [29,30,31]. PMA is established to induce a fast and transient block of Cx43 gap junctional communication in many cell types [32,33,34,35]. This is often followed by the accelerated endocytosis and degradation of Cx43 gap junctions [36,37]. PMA also blocks this assembly of gap junctions [38]. The immunofluorescence assay revealed that the treatment of MDA-Cx43D with PMA affects Cx43 localization and induces the formation of annular junctions as early as 2 h, and this effect was sustained 24 h post-PMA treatment (Figure 1D). Moreover, co-immunoprecipitation, 2 or 24 h post-PMA induction, indicates that Cx43 URMylation and SUMOylation by SUMO-1 occur as early as 2 h and increase upon exposure to PMA (Figure 1E). This result demonstrates that Cx43 internalization is accompanied by PTMs. MCF-7 cells were also exposed to PMA and analyzed for Cx43 and URM-1 levels. Western blot analysis showed decreased Cx43 levels 3 h post-PMA treatment and increased URM-1 levels within 1 h of PMA treatment, which subsequently decreased (Supplementary Figure S1B). Collectively, these results indicate that URM-1 can interact with and modify Cx43, similar to other ubiquitin-like small protein modifiers.

### 2.2. Downregulation of URM-1 Modulates Cx43 Expression

**Figure 2:**
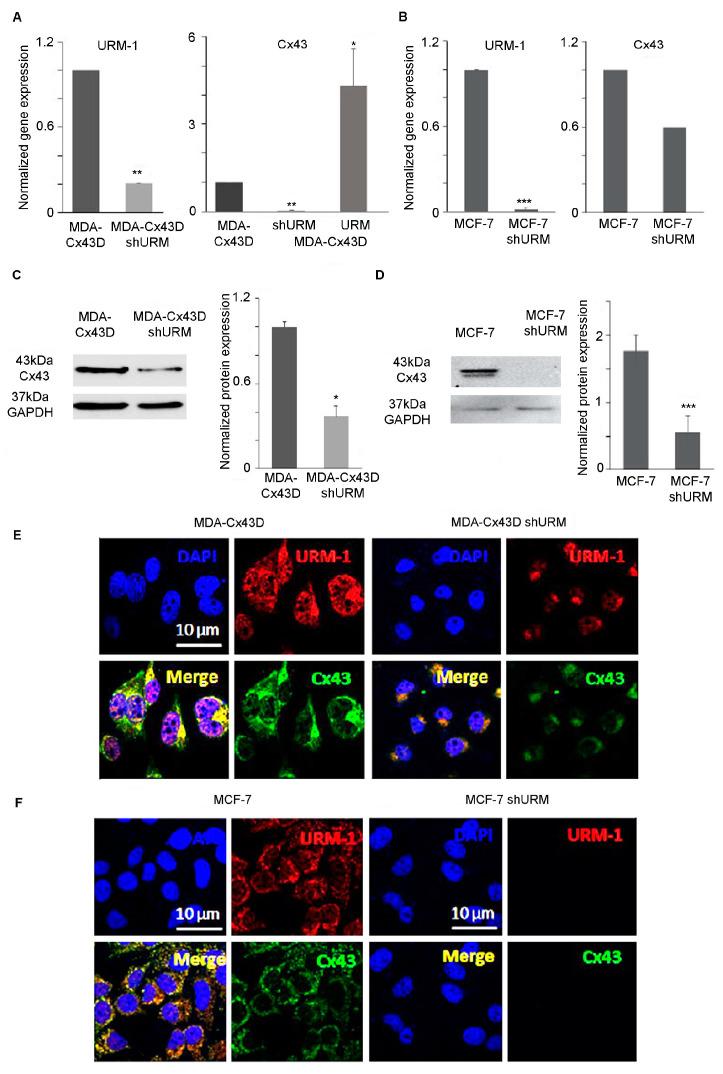
Downregulation of URM-1 regulates Cx43 in breast cancer cells. (**A**) Bar graphs display normalized mRNA expression levels of URM-1 and Cx43 in MDA-Cx43D, MDA-Cx43D shURM, and MDA-Cx43D URM, as detected by qPCR. Downregulation of URM-1 resulted in significantly decreased Cx43 transcript levels, and upregulation of URM-1 resulted in significantly increased Cx43 transcript levels. (**B**) Bar graphs show normalized gene expression levels of URM-1 and Cx43 in MCF-7 and MCF-7 shURM cells. Cells downregulating URM-1 also downregulate Cx43, though this decrease did not reach the statistical significance threshold. (**C**) Western blot of Cx43 expression in MDA-Cx43D and MDA-Cx43D shURM, with densitometry analysis after normalization to GAPDH, shows significantly decreased Cx43 protein levels in cells downregulating URM-1. (**D**) Protein levels of Cx43 are significantly downregulated in MCF-7 shURM cells, as shown by Western blot and densitometry. (**E**) Representative immunofluorescence images of Cx43 and URM-1 expression in MDA-Cx43D and MDA-Cx43D shURM. (**F**) Representative immunofluorescence images of Cx43 and URM-1 expression in MCF-7 and MCF-7 shURM cells. Results of qPCR and Western blotting are representative of three independent experiments and are displayed as averages ± SD. Immunofluorescence was performed twice, and the micrographs are representative images of several fields from at least two independent experiments. * *p* < 0.05, ** *p* < 0.01, and *** *p* < 0.001.

The URMylation of Cx43 occurs as early as 2 h upon cellular stress. We assessed whether changes in URM-1 expression modulate the Cx43 cellular distribution. The downregulation of URM-1 in MDA-Cx43D and MCF-7 cells decreased URM-1 transcript levels by 80% (*p* < 0.01) and 100% (*p* < 0.001), respectively (Figure 2A,B). This downregulation was also verified by Western blot analysis (Supplementary Figure S1D). Conversely, upregulated URM-1 in MDA-Cx43D cells (MDA-Cx43D URM) resulted in upregulated Cx43 transcript levels (Figure 2A). MDA-Cx43D shURM and MCF-7 shURM cells also showed significantly decreased translational levels of Cx43 (Figure 2C, *p* < 0.05 and 2D, *p* < 0.001). Immunofluorescence analysis demonstrated that URM-1 co-localizes with Cx43 (Figure 2F). Importantly, the downregulation of URM-1 affected the expression of Cx43 in MDA-Cx43D shURM and MCF-7 shURM cells (Figure 2E,F).

### 2.3. Loss of URM-1 Promotes EMT in Breast Cancer Cell Lines

**Figure 3:**
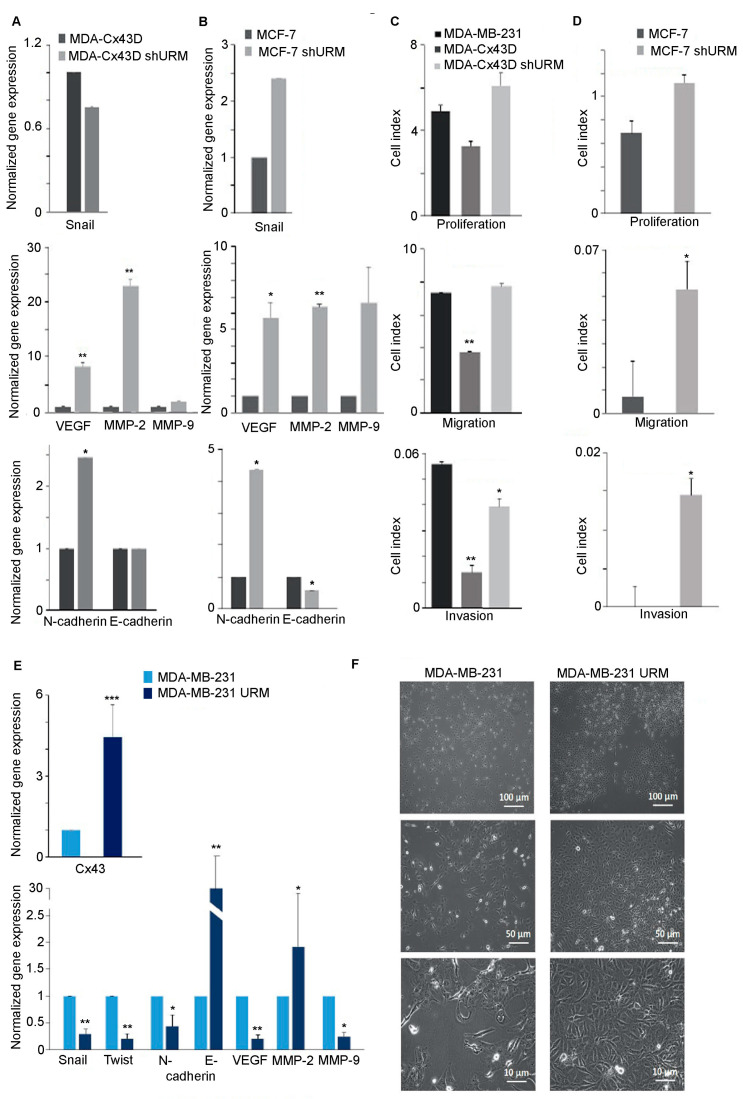
Loss of URM-1 promotes EMT in breast cancer cell lines. (**A**) Transcriptional levels of Snail, VEGF, MMP-2, MMP-9, and N- and E-cadherin in MDA-Cx43D and MDA-Cx43D shURM. VEGF and MMP-2 significantly increased in MDA-Cx43D shURM cells, as shown by qPCR. These cells also expressed higher levels of mesenchymal marker N-cadherin. E-cadherin levels remained stable in MDA-Cx43D shURM cells. (**B**) Transcriptional levels of Snail, VEGF, MMP-2, MMP-9, and N- and E-cadherin in MCF-7 and MCF-7 shURM. VEGF, MMP-2, and N-cadherin significantly increased in MCF-7 shURM. MMP-9 levels also increased, but this increase did not reach statistical significance. E-cadherin levels decreased in MCF-7 shURM cells. (**C**,**D**) RTCA was performed to evaluate proliferation, migration, and invasion potential of breast cancer cells downregulating URM-1. MDA-Cx43D shURM and MCF-7 shURM cells displayed enhanced proliferation (although differences did not reach statistical significance), migration, and invasion, compared to their URM-1-expressing counterparts. Bar graph displays the cell index recorded for all experimental cells. The higher the cell index, the higher the proliferation, migration, or invasion activity. (**E**) MDA-MB-231 cells were then upregulated with URM-1. Bar graphs show significantly increased levels of Cx43 in MDA-MB-231 URM cells, decreased transcriptional levels of mesenchymal markers Snail, twist, N-cadherin, VEGF, and MMP-9, and a major increase in epithelial marker E-cadherin. (**F**) Light microscopy images also show changes in morphology, consistent with an epithelial-to-mesenchymal transition of breast cancer cells upregulating URM-1. qPCR results are representative of three independent experiments and are displayed as averages ± SD. RTCA experiments have been run twice: proliferation (in triplicate), migration, and invasion (in duplicate), * *p* < 0.05, ** *p* < 0.01 and *** *p* <0.001.

Given the tumor suppressor role of Cx43 in breast cancer and the apparent modulation of Cx43 by URM-1 levels in breast cancer cell lines, cells were evaluated for changes in the levels of EMT markers upon URM-1 downregulation. The transcriptional levels of Snail, VEGF, MMP-2, MMP-9, N-cadherin, and E-cadherin were evaluated by qPCR. Gene expression analysis showed that MDA-Cx43D shURM cells expressed significantly higher levels of VEGF and MMP-2 (*p* < 0.01, Figure 3A). The levels of Snail, VEGF, and MMP-2 were also significantly increased in MCF-7 shURM cells (*p* < 0.05, *p* < 0.05, and *p* < 0.01, respectively, Figure 3B). Both MDA-Cx43D shURM and MCF-7 shURM cells expressed higher levels of MMP-9 and the mesenchymal marker N-cadherin and did not change or decreased the level of the epithelial marker E-cadherin, but these changes did not reach the statistical significance threshold (Figure 3A,B). Functionally, these findings were evaluated using a real-time cell analyzer (RTCA). The proliferation, migration, and invasion potential increased among cells downregulating URM-1, as reflected by the increased cell index. In particular, the loss of URM-1 rendered MDA-Cx43D shURM cells significantly more invasive (*p* < 0.05, Figure 3C) and significantly increased the migration and invasion capacity of MCF-7 shURM cells (*p* < 0.05, Figure 3D). URM-1 upregulation in MDA-MB-231 cells was reflected by a parallel increase in the Cx43 expression level in MDA-MB-231 URM cells (*p* < 0.001, Figure 3E). The evaluation of EMT markers in these cells by qPCR showed that upon the upregulation of URM-1, the transcript levels of Snail, Twist, and VEGF (*p* < 0.01) and of N-cadherin and MMP-9 (*p* < 0.05) significantly decreased (Figure 3E). In addition, the mRNA levels of the epithelial marker E-cadherin greatly increased (*p* < 0.01) (Figure 3E). These changes translated to morphological alterations; light microscopy images show that MDA-MB-231 cells upregulating URM-1 display a less mesenchymal phenotype (fibroblastic in appearance and present as spindle-shaped flattened cells) and a more epithelial phenotype (monolayers with sheet-like appearance) (Figure 3F).

**Figure 4:**
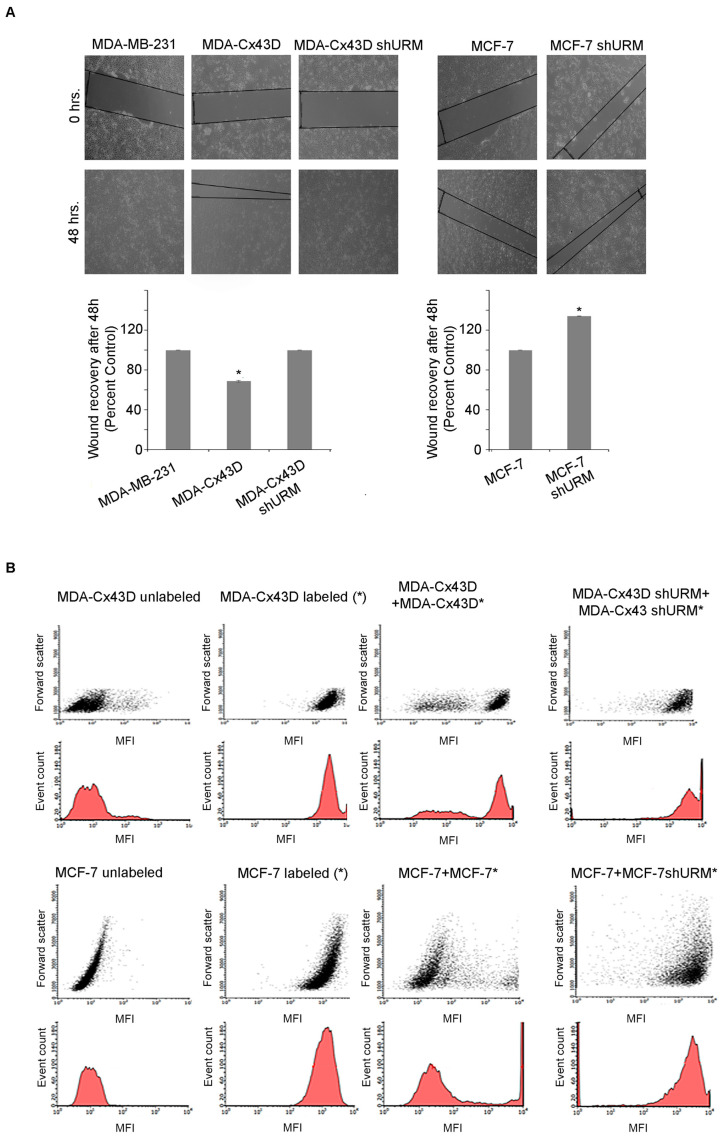
Loss of URM-1 affects the capacity of Cx43 to form functional gap junctions in breast cancer cell lines. (**A**) Cell migration determined by wound healing assay: Upper panel shows optical microscope images presenting scratch areas covered by migrating MDA-MB231, MDA-Cx43D, MDA-Cx43D ShURM, MCF-7, and MCF-7 just after scratching (0 h) and 48 h after scratching. Lower panel shows quantitative evaluation of wound closure (%) expressed as the percentage of the wound area covered by the cells 48 h after scratching. 10× magnification. (**B**) Flow cytometry analysis of dye transfer assay showing gap junction intercellular communication established between MDA-Cx43D, labeled MDA-Cx43D (MDA-Cx43D*), and MDA-Cx43D ShURM* or MCF-7 and MCF-7 ShURM*. Forward scatter, cell count, and mean fluorescence intensity are represented in the graphs. The depicted result is a representative experiment of three independent ones.

To determine the implication of Cx43 URMylation on the functionality of connexins in forming functional gap junctions between breast cancer cells, two modalities were adopted: the in vitro wound healing assay and dye transfer. In the first modality, we examined cell migration in response to a mechanical scratch wound in the presence or absence of URMylated Cx43. Images of scratch areas at time points of 0 and 48 h are illustrated in Figure 4A. While the scratch was completely closed after 48 h in MDA-MB-231, the overexpression of Cx43 in these cells induced the partial recovery of the scratch, implicating Cx43 in the migration capacity of MDA-MB-231. Interestingly, URM downregulation in MDA-Cx43D restored the migration capacity of these cells, and the scratch completely closed, as in the parental MDA-MD-231 at 48 h (Figure 4A). In MCF-7 cells, the recovery of the scratch was partial, but the downregulation of URM significantly increased wound recovery after 48 h (*p* < 0.05) (Figure 4A). In the dye transfer assay, calcein-labeled MDA-Cx43D or MDA-Cx43D ShURM cells were co-cultured with unlabeled MDA-Cx43D or MDA-Cx43D ShURM, respectively, at a 1:1 ratio for 1 h. Similarly, labeled MCF-7 or MCF-7 ShURM cells were co-cultured with unlabeled MCF-7; then, dye transfer between the tested cells was evaluated by flow cytometry, and the mean fluorescence intensity (MFI) was quantified. We observed a clear shift in fluorescence intensity in both labeled breast cancer cell lines (Figure 4B). The downregulation of URM in either cell line reduced or blocked the shift in fluorescence, emphasizing that dye transfer occurred through URMylated Cx43 forming gap junctions (Figure 4B). Collectively, these data implicate URM-1 in the breast cancer cell malignant phenotype, presumably due to the loss of Cx43 in cells downregulating URM-1. The upregulation of URM-1 seems to, at least partially, reverse EMT in breast cancer cells. Moreover, the URMylation of Cx43 is required for its functionality and the formation of functional gap junctions.

## 3. Discussion

Post-translational modifications modulate protein expression, assembly, and trafficking and determine their definitive functions [39]. The connexin family of proteins, including Cx43, is post-translationally modified, leading to the opening or closing of the channel or insertion into specialized lipid rafts, which induces dramatic functional changes [40]. Since Cx43 is modified by ubiquitylation [41,42] and SUMOyaltion [43], we explored whether it is also modified by another ubiquitin-like molecule, URM-1, known to rapidly conjugate to multiple target proteins in response to oxidative stress [41,42,43].

We demonstrated that Cx43 is modified with URM-1, Ub, and SUMO-1, but not SUMO-2/3, in MCF-7 and MDA-231-Cx43D. This is in accordance with several studies showing that connexins can be ubiquitylated on specific lysine residues (K9 and K303), leading to the proteasomal degradation of connexins [41]. In addition, Cx43 undergoes post-translational modifications by less characterized pathways, such as SUMOylation [43]. In fact, in MDA-MB-231 and MCF-7 cell lines, Cx43 is SUMOylated by SUMO-1, but not SUMO-2/3. Another study showed that Cx43 may be modified by SUMO-1/2/3 in human cervical cancer cells, HeLa-CCL2 transfected with Cx43 [43], indicating that Cx43 SUMOylation could be cell-type-dependent.

After demonstrating the co-localization of Cx43 with Ub, URM-1, and SUMO-1 in MDA-Cx43D and MCF-7 cells and the interaction of Cx43 with each of these PTMs, the effect of PMA-induced stress was investigated in an attempt to determine a sequence of post-translational modifications of Cx43 or an interplay between ubiquitylation, SUMOylation, and URMylation. MDA-Cx43D cells were exposed to PMA, which induces cellular stress through the PKC-dependent internalization of Cx43 and hence hinders Cx43-mediated intercellular communication [29,30]. Enhanced URMylation by URM-1 was followed by enhanced SUMOylation by SUMO-1 upon PMA treatment, in line with a previous study reporting a substantial increase in SUMO conjugation upon exposure to stress [44]. Collectively, our data suggest that the URMylation of Cx43 by URM-1 may be a prerequisite to SUMOylation by SUMO-1. In fact, a study showed that mutations in the lysine residues of an oncoprotein, Tax, compromise its URMylation by URM-1 [28]. It is conceivable that the URMylation of Cx43 occurs on conserved lysine residues, which requires further investigation.

Cells downregulating URM-1 showed lower transcriptional and translational levels of Cx43. Connexins in general and the loss of Cx43 in particular are closely associated with cancer progression and metastasis [7,8,10,16,21,23]. We have previously shown that upregulating Cx43 in MDA-MB-231 attenuates its malignant phenotype [7]. Since downregulating URM-1 was associated with decreased levels of Cx43, we investigated EMT markers in MDA-Cx43D shURM and MCF-7 shURM cell lines. The levels of VEGF, MMP-2, and MMP-9 were upregulated in these cell lines compared to their Cx43-expressing counterparts. These findings translated to functional alterations, with the loss of URM-1 (associated with the loss of Cx43) enhancing the proliferation, migration, and invasion profiles of breast cancer cells. Importantly, while MCF-7 cells are usually poorly invasive, the loss of URM-1 enhanced their malignant profile. In addition, upregulating URM-1 was associated with the significant and considerable reversal of EMT, inducing MDA-MB-231 cells to acquire a more epithelial phenotype.

Collectively, our study unveils a novel interaction of URM-1, a ubiquitin-related modifier, with Cx43, a widely recognized gap junction protein with a tumor suppressor role in breast cancer, and demonstrates that the loss of URM-1 promotes EMT, while the upregulation of URM-1 drives EMT reversal in breast cancer.

## 4. Materials and Methods

### 4.1. Cells and Cell Cultures

Wild-type (parental) triple-negative breast adenocarcinoma epithelial MDA-MB-231 cells overexpressing Cx43 (MDA-Cx43D) were generated as previously described [7]. Briefly, pDendra2-N plasmid was purchased from Evrogen (Russia) and used to generate a pCSCW-Dendra2-Cx43 lentiviral vector. The latter was co-transfected with packaging plasmids, using the calcium phosphate method, into HEK293T cells for the production of viral particles that were collected from the supernatant 48 and 72 h post-transfection. MDA-MB-231 cells were then transduced with lentiviral particles to generate MDA-MB-231 cells overexpressing Cx43 (referred to as MDA-Cx43D) and sorted using FACS Aria III SORP (BD Biosciences, Heidelberg, Germany). Luminal MCF-7 cells were also used in this study. URM-1 was downregulated using the lentiviral shURM vectors in MDA-Cx43D and MCF-7 cells to generate MDA-Cx43D shURM and MCF-7 shURM cells. Knockdown of URM-1 was performed by transfecting cells using the urm-1 shURM plasmid (cat# sc-92844-SH, Santa Cruz, CA, USA) obtained from Abgent (San Diego, CA, USA). In the overexpression experiments, URM-1 was upregulated using the pcDNA4.0:URM-1 plasmid (cat# DC01337, Abgent, San Diego, CA, USA). Transfections were performed using Lipofectamine 2000^TM^ (ThermoFisher Scientific, Waltham, MA, USA) [45], and MDA-Cx43D, MDA-Cx43D shURM, and MCF-7 shURM were generated. All cells were maintained in RPMI-1640 medium (Sigma, St Louis, MO, USA) supplemented with 10% fetal bovine serum (FBS, Sigma, St Louis, MO, USA) and penicillin/streptomycin (100 units of potassium penicillin and 100 μg of streptomycin sulfate per 1 mL of medium, Sigma, St Louis, MO, USA). Phorbol 12-myristate13-acetate (PMA, Sigma, Missouri, USA) was used at a concentration of 100 ng/mL to induce cellular stress by internalizing Cx43 and inhibiting Cx43-mediated intercellular communication [33].

### 4.2. Analysis of Gene Expression

RNA extraction and reverse transcription. Total RNA was extracted with RNeasy^®^ Plus mini kit (Qiagen, Germany) according to the manufacturer’s instructions and served as the template for cDNA synthesis with Random Hexamers and SuperScript^®^II Reverse Transcriptase (ThermoFisher Scientific, MA, USA).

Gene expression analysis. Quantitative real-time polymerase chain reaction (qPCR) was performed in a CFX96™ Real-Time PCR Detection System (Bio-Rad, Hercules, California, USA) using the qPCR Sybr green Master Mix. Primer sequences are listed in Table 1. The relative fold change in gene expression was calculated using the ΔΔ^Cq^ method after normalization to the housekeeping gene *gapdh* [46].

### 4.3. Analysis of Protein Expression and Localization

Antibodies used for protein detection. Primary antibodies used in the different assays were all raised against human antigens, as follows: mouse URM-1 (A-7) antibody (cat# sc-374485, lot# A0814, Santa Cruz, TX, USA), rabbit Ub (F-11) antibody (cat# sc-271289, lot# B0316, Santa Cruz), mouse SUMO-1 (D-11) antibody (cat# sc-5308, lot# K1011, Santa Cruz), rabbit SUMO 2-3 (FL-103) antibody (cat# sc-32873, lot# J2010, Santa Cruz), α-GAPDH clone B2534M antibody (cat# MAB547, Abnova, CA, USA), and two Cx43 antibodies (goat anti-Cx43 C-20, cat# sc-6560, lot# F259, Santa Cruz; and rabbit anti-Cx43 cat# SAB4501175, lot# 310158, Sigma, Mannheim, Germany). Secondary antibodies used for Western blotting were mouse anti-rabbit IgG-HRP (cat# sc-2357, Santa Cruz) and goat anti-mouse IgG-HRP (sc-2031, Santa Cruz); for immunofluorescence, Texas Red goat anti-mouse- or goat anti-rabbit- or Alexa Fluor 488 donkey anti-goat-conjugated secondary antibodies (Invitrogen, Carlsbad, CA, USA) were used.

Immunofluorescence. Immunofluorescence staining was performed on cells seeded onto glass coverslips, as previously described [25]. Antibodies against URM-1, rabbit Cx43, SUMO-1, SUMO2/3, and Ub F-11 were added to the cells and incubated overnight. Cells were then incubated with Texas Red- or Alexa Fluor 488-conjugated secondary antibodies. Nuclear staining was performed using 1 μg/mL 4′,6-Diamidino-2-Phenylindole (DAPI) dye. Coverslips were mounted onto glass slides using Prolong Anti-fade reagent (Invitrogen, Carlsbad, CA, USA).

In situ proximity ligation assays (Duolink^®^, Shinagawa City, Tokyo). Cells were seeded onto glass coverslips, fixed, and permeabilized in ice-cold methanol. Protein–protein interactions were visualized using the Duolink^®^ in situ proximity ligation assay (PLA) system (Olink Bioscience, Uppsala, Sweden) using antibodies against SUMO-1, SUMO-2/3, Ub, Cx43, and URM-1.

Co-immunoprecipitation. Cells were washed in ice-cold phosphate-buffered saline (PBS) supplemented with 10 mM N-ethylmaleimide (NEM) prior to lysis at pH 8 in 2% SDS and 50 mM Tris. Lysates were then 10-fold diluted in an IP buffer comprising 50 mM Tris-HCl (pH 8), 200 mM NaCl, 0.1 mM EDTA, 0.5% NP-40, 10% glycerol, and a cocktail of protease inhibitors (Roche). Afterward, 1 µg of goat anti-human Cx43 (C-20) was added for 12 h at 4 °C, followed by the addition of protein A-agarose (Sigma, Mannheim, Germany) for 2 h. Beads were washed in IP buffer three times prior to elution of immunoprecipitated proteins. The latter were then solubilized in 2× Laemmli buffer and denatured using β-mercaptoethanol (5%), followed by heating at 95 °C for 10 min. Proteins were then loaded onto a 10% polyacrylamide gel for Western blotting.

Western blotting. Proteins were extracted in lysis buffer (126 mM Tris/HCl, 20% glycerol (*v*/*v*), and 40 mg/mL of sodium dodecyl sulfate (SDS)) in the presence of phosphatase (PhosSTOP, cat# 04906846001, Roche Diagnostics GmbH, Mannheim, Germany) and protease inhibitors (cOmplete^TM^, cat# 11836153001, Roche Diagnostics GmbH, Mannheim, Germany). Protein concentrations were determined using DC Protein Assay II kit (Bio-Rad, Feldkirchen, Germany). Total protein lysates (100 µg) were resolved by SDS-PAGE on a 10% polyacrylamide gel and then transferred to PVDF membranes (Bio-Rad, Hercules, CA, USA), which were then blocked with 5% skimmed milk for 1 h before incubation with primary antibodies at a concentration of 1 µg/mL against SUMO-1, Cx43, and URM-1 (Santa Cruz, TX, USA).

### 4.4. Imaging

Cell morphology and confluence were observed, and images were acquired using Cell Observer and Zen software (Zen Lite 2009, Carl Zeiss, Germany). Immunofluorescence micrographs were visualized and imaged using a Laser Scanning Confocal Microscope (LSM 710, Carl Zeiss, Oberkochen, Germany).

### 4.5. Real-Time Cell Analyzer (RTCA) Assay for Migration, Invasion, and Proliferation

To assess the effect of URM-1 loss on migration, invasion, and proliferation of cell lines, quantitative analysis was performed using RTCA (×CELLigence RTCA[A2]DP, Roche, Penzberg, Germany), as previously described [47]. For migration and invasion assays, cells were re-suspended in serum-free media and seeded at a density of 20,000 cells/well of RTCA CIM-plates coated with Matrigel^®^ for the invasion assay and without Matrigel^®^ for the migration assay. Cells were allowed to adhere for 24 h. Invasion was monitored by recording cell impedance every 15 min for a minimum of 18 h. For the proliferation assay, cells were seeded in an E-plate at a density of 7000 cells/well with an additional 120 μL of medium containing 10% serum. Proliferation was monitored by recording cell impedance every 60 min for 24 h. Experiments were performed twice in triplicate for the proliferation assay and in duplicate for migration and invasion assays. The RTCA software generated a survival curve that evaluates the cell survival or cell index (CI). CI directly correlates with cell number. Data were expressed as bar graphs of CI % of control.

### 4.6. In vitro Wound Healing Assay

MDA-MD231, MDA-Cx43D, MDA-Cx43D ShURM, MCF-7, or MCF-7 ShURM cells (3 × 10^5^ cells/well) were seeded in 24-well plates to grow in a monolayer for 24 h. Then, a sterile 20–200 μL pipette tip was held vertically to scratch a cross in each well. The detached cells were removed by washing with 500 μL of PBS and shaking at 500 rpm for 5 min. Afterward, 500 μL of fresh medium with or without diluted samples was added and incubated for 72 h. Before image acquisition, the plate was washed with 500 μL of pre-warmed PBS and gently shaken for 30 s. Then, the pre-warmed medium or sample was added again, and pictures were taken. The scratch closure was monitored and imaged in 24 h intervals. The analysis of the scratch images was performed using ImageJ. The percentage of open wound area was plotted over time for each concentration. Data are presented as mean ± SD. Three to six replicates were included in the analysis, and an unpaired Student’s *t*-test was performed. Significance was considered at *p* < 0.05.

### 4.7. Dye Transfer Assays

MCF-7, MCF-7 ShURM, MDA-Cx43D, and MDA-Cx43D ShURM were seeded in 6-well plates at a density of 25 × 10^3^ per cm^2^ for 24 h. Cells were labeled with 1 μM Calcein for 1 h, washed, and incubated with serum-free medium for 30–60 min to allow intercellular esterase to convert non-fluorescent Calcein to a green-fluorescent form. Labeled MCF-7 or MCF-7 ShURM cells were co-cultured with unlabeled MCF-7. Similarly, labeled MDA-Cx43D or MDA-Cx43D cells were co-cultured with unlabeled MDA-Cx43D for 1 h. The co-cultures were incubated in complete media at 37 °C and 5% CO_2_ for 1 h. Non-adherent cells were then removed by washing, and the adherent cells were detached by trypsinization and re-suspended in phosphate-buffered saline (PBS) containing 2% formaldehyde to be analyzed by flow cytometry. Dye transfer was evaluated by measuring mean fluorescence intensity (MFI). Mean fluorescence intensities of both the labeled and unlabeled populations were evaluated using dot plots based on fluorescence of unlabeled and calcein-labeled control samples.

### 4.8. Statistical Analysis

Experiments were performed three times, unless specified otherwise in the figure legends. Results of quantitative data are reported as the mean ± standard deviation (SD). Statistical significance was determined using Student’s *t*-test. The *p* value was determined, and a *p* value of 0.05 was set as the significance threshold. Microsoft Excel was used for statistical analysis.

## Figures and Tables

**Table 1:** qPCR primer sequences.

Gene	Primer Sequences	Annealing Temperature
Cx43	F: CTTCACTACTTTTAAGCAAAAGAG R: TCCCTCCAGCAGTTGAG	52 °C
GAPDH	F: TGGTGCTCAGTGTAGCCCAG R: GGACCTGACCTGCCGTCTAG	52–62 °C
URM-1	F: GGGCGGAGTTACTATTTGGT R: TCATAACCGATTTCACTCAAGTTT	57 °C
MMP-2	F: TTGACGGTAAGGACGGACTC R: ACTTGCAGTACTCCCCATCG	55 °C
MMP-9	F: TTGACAGCGACAAGAAGTGG R: GCCATTCACGTCGTCCTTAT	55 °C
VEGF	F: AGGCCCACAGGGATTTTCTT R: ATCAAACCTCACCAAGGCCA	55 °C
N-cadherin	F: GGTGGAGGAGAAGAAGACCAG R: GGCATCAGGCTCCACAGT	58 °C
E-cadherin	F: CAGAAAGTTTTCCACCAAAG R: AAATGTGAGCAATTCTGCTT	58 °C
Snail	F: CTTCCAGCAGCCCTACGAC R: CGGTGGGGTTGAGGATCT	58 °C
Twist	F: AGCTACGCCTTCTCGGTCT R: CCTTCTCTGGAAACAATGACATC	58 °C

Cx43: connexin 43; F: forward sequence; GAPDH: glyceraldehyde 3-phosphate dehydrogenase; MMP: matrix metalloproteinase; R: reverse sequence; URM-1: ubiquitin-related modifier 1; VEGF: vascular endothelial growth factor.

## Data Availability

Raw data are available upon reasonable request.

## Supplementary Materials

The following supporting information can be downloaded at: https://www.mdpi.com/article/10.3390/ijms24032958/s1.
