# Supplementary material for: Ubiquitin-Related Modifier 1 (URM-1) Modulates Cx43 in Breast Cancer Cell Lines
Source: Int J Mol Sci. 2023 Feb 3;24(3):2958. doi: 10.3390/ijms24032958 (PMC9917400; doi:10.3390/ijms24032958)
Supplement: Supplementary file 1 [file ijms-24-02958-s001.zip › ijms-2059408-supplementary.pdf]

Figure S1

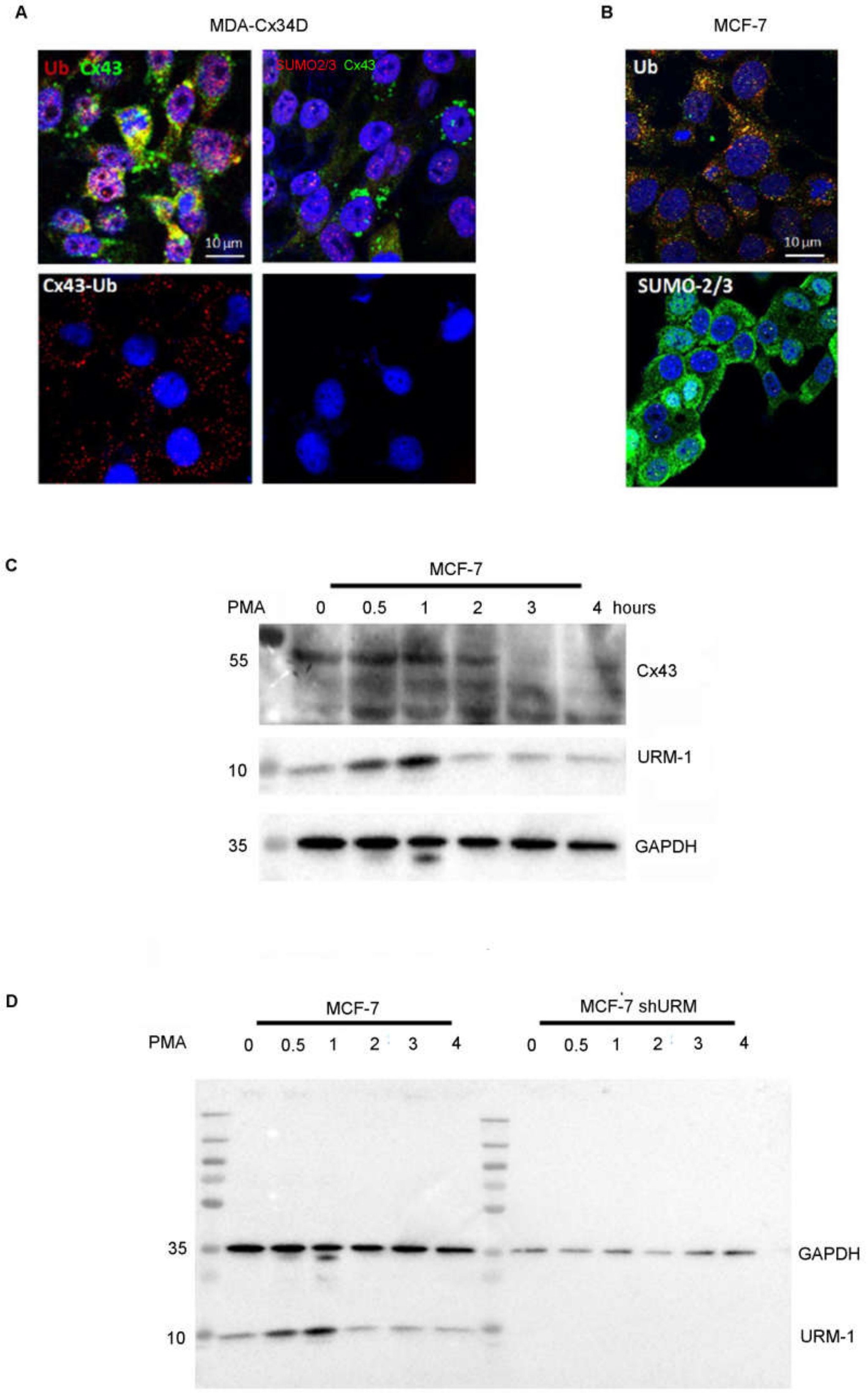

Figure S1. Cx43 interacts with ubiquitin, but not with SUMO2/3 in breast cancer cells. A. Immunofluorescence assay (upper panel) and Duolink® in situ proximity ligation assay (lower

panel) were performed in MDA-Cx43D cells. Cx43 co-localizes and interacts with Ub, primarily in the cytoplasmic compartment. MDA-Cx43D cells do not express SUMO-2/3 and no colocalization with SUMO2/3 was observed (left panel). **B.** Immunofluorescence assay shows co-localization of Cx43 with Ub, but not with SUMO2/3 in MCF-7 cells. DAPI is shown in blue, SUMO-1, SUMO-2/3, Ub and URM are shown in red and Cx43 in green. **C**. MCF-7 cells were also exposed to PMA and analyzed for Cx43 and URM-1 levels. Western blot analysis showed decreased Cx43 levels 3 hours post-PMA treatment and increased URM-1 levels within 1 hour of PMA addition, to subsequently decrease. Experiment shown in panel C was done once. **D**. MCF-7 or MCF-7 shURM cells were exposed to PMA and analyzed for URM-1 levels. Western blot analysis showed a clear downregulation of URM-1 levels at all tested conditions. Experiment shown in panel D was done once.
